# Manual of Peripheral Nerve Surgery

**DOI:** 10.1055/s-0038-1669393

**Published:** 2018-08-13

**Authors:** J. Bahm

**Affiliations:** 1 Euregio Reconstructive Microsurgery Unit, Franziskus Hospital, Aachen, Germany

**Manual of Peripheral Nerve Surgery edited by M. Socolovsky, L. Rasulic, R. Midha, and D. Garozzo [Thieme 2018]**

This concise and well-focused compendium is the fruit of a very active Peripheral Nerve Surgery Committee within the World Federation of Neurosurgical Societies, driven by the book editors who represent clinical and scientific knowledge in this particular field from different continents—a must in an area with sometimes rare clinical cases and few dedicated specialists in every country.

The present book is perfect for beginners or experts and is based on solid ground, starting with current reviews on nerve anatomy of the limbs, the clinical aspect of nerve trauma including war injuries, and diagnostic tools (electrodiagnosis, magnetic resonance, and ultrasound).

The different neurosurgical techniques are detailed in this book, starting with neurolysis, direct repair, nerve grafting, and increasing use of tubes.

Compressive lesions including the thoracic outlet syndrome are presented; several chapters deal with the traumatic brachial plexus lesions, in adults and the neonate, and lumbosacral plexus.

Reconstruction of the facial nerve palsy is exposed by one skilled neurosurgeons' personal experience and the last two chapters summarize the actual knowledge on benign and malignant peripheral nerve tumors, wherein many authors contributed their knowledge and cases together to help readers better understand the guidelines on diagnosis and surgical strategy.

The editors stand for an excellent activity in their committee, aimed to raise interest and performance in the sometimes “neglected” field of the peripheral nerve, also addressed in other surgical specialties such as plastic and hand or orthopaedic surgery.

Dedication to the peripheral nerve is a surgical passion, and the book reflects this enthusiasm. Although many textbooks on this topic came up in the last decade, this work is a milestone as it has a clear message as stated in the title “from the basics to complex procedures” in ∼200 pages, allowing the newcomers to go through it in a reasonable time without being discouraged by a 2,000 page encyclopedia, and still detailed enough on so delicate topics like the malignant peripheral nerve sheath tumors to satisfy surgical experts, facing rare and complex cases.

Also, it is not just about “doing”: a lot of emphasis is given to the timing and the outcome of nerve reconstructions, which are not always perfect or predictable and frequently claim an interdisciplinary approach with secondary procedures performed by other specialties. But this is beyond the scope of this book, which clearly fulfils its scope, to address all those, especially neurosurgeons and neurologists, who want to know how surgery on peripheral nerves should be indicated, done, and followed up.

This manual fits in the list of existing textbooks as being very well documented, clearly written, and concise, reflecting today's peripheral nerve surgeon's activity.

